# Restoring energy metabolism by NAD ^+^ supplement prevents alcohol‐induced liver injury and boosts liver regeneration

**DOI:** 10.1002/fsn3.4159

**Published:** 2024-04-22

**Authors:** Yao Liu, Cheng Cheng, Han Gao, Xue‐jin Zhu, Xian He, Ming‐xi Zhou, Yuan Gao, Ya‐wen Lu, Xin‐hua Song, Xiao‐he Xiao, Jia‐bo Wang, Chun‐jun Xu, Zhi‐tao Ma

**Affiliations:** ^1^ School of Traditional Chinese Medicine Capital Medical University Beijing China; ^2^ Department of Infectious Disease, Beijing Hospital of Traditional Chinese Medicine Capital Medical University Beijing China; ^3^ Department of Pharmacy Jincheng General Hospital Jincheng Shanxi China; ^4^ College of Pharmacy Fujian University of Traditional Chinese Medicine Fuzhou China; ^5^ Department of Hepatology Fifth Medical Center of Chinese PLA General Hospital Beijing China

**Keywords:** alcoholic liver disease, energy, liver regeneration, metabolism, mitochondria, nicotinamide

## Abstract

Our previous clinical metabolomics study illustrated that energy metabolism disorder is an underlying pathogenesis mechanism for the development of alcoholic liver disease (ALD). Supplementation of nicotinamide (NAM), the precursor of nicotinamide adenine dinucleotide (NAD^+^), may restore the energy metabolism homeostasis of ALD and thus serves as potential therapeutics to treat ALD. In this bedside‐to‐bench study, the protective effect of NAM against ALD was investigated by using the NIAAA mice model (chronic‐plus‐binge ethanol), and the liver regeneration boosting capability of NAM was evaluated by the partial hepatectomy mice model. Our results showed that NAM supplements not only protected the liver from alcohol‐induced injury and improved alcohol‐induced mitochondrial structure and function change, but also boosted liver regeneration in postpartial hepatectomy mice by increasing liver NAD^+^ content. These findings suggested that NAM, a water‐soluble form of vitamin B3, can promote liver regeneration and improves liver function by alleviating alcohol‐induced energy metabolism disorder.

## Graphical abstract

Nicotinamide can improve liver function and repair the structure and function of mitochondria in chronic‐plus‐binge alcohol‐feeding mice. Nicotinamide can significantly improve alcohol‐induced energy metabolism disorder. Nicotinamide promotes liver regeneration in mice receiving partial hepatectomy. Nicotinamide would be promising therapeutics for alcoholic liver disease.

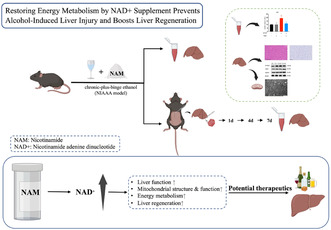

## 1 INTRODUCTION

Long‐time or heavy alcohol consumption, without a doubt, contributes to liver injury, leading to alcoholic liver disease (ALD) (Seitz et al., 2018; Singal et al., 2018). The pathological change of ALD generally starts from hepatic steatosis, followed by hepatitis or fibrosis, eventually progressing to cirrhosis, which ultimately may drive to hepatocellular carcinoma (Altamirano & Bataller, 2011; Gao & Bataller, 2011). Despite each stage of alcohol‐induced liver injury being well characterized, there is no consensus curative or preventive therapy available at present (Singal & Mathurin, 2021). Given the situation, there is increasing attention on investigating the biochemical changes of the development and progression of ALD, aiming at developing a novel therapy for ALD prevention or treatment.

Previously, we did sera untargeted metabolomics assay on ALD patients from different clinical stages, and the results showed that the abnormal activation of lipogenesis and proteolysis is the major metabolic characteristic of ALD progress (Cheng et al., 2022; Huang et al., 2021). The therefrom energy synthesis and supply deficiency suppress both normal liver function and the postinjury repair capacity, which might be a dual strike for the alcoholic liver. This finding leads to an interesting hypothesis: could modifying energy metabolism be a potential approach to prevent or treat ALD? Nicotinamide adenine dinucleotide (NAD^+^), a key regulator of numerous energy metabolism pathways including glycolysis, tricarboxylic acid (TCA) cycle, and mitochondrial respiration (Chini et al., 2021; Covarrubias et al., 2021; Fouquerel & Sobol, 2014; Verdin, 2015), is converted to NADH in large amounts during chronic ethanol intake, lowering the NAD^+^/NADH ratio; and thereby inhibiting mitochondrial oxidative phosphorylation and energy metabolism (Cantó et al., 2015; Comporti et al., 2010). Considering all the above, we propose the assumption that NAD^+^ supplement might be a strategy to prevent or treat ALD by modifying energy metabolism.

Nicotinamide (NAM), a water‐soluble form of vitamin B3, widely exists in our daily diets and is usually taken as a food supplement to keep fit (Chini et al., 2021; Yoshino et al., 2018). NAM serves as a precursor of NAD^+^ (Zapata‐Pérez et al., 2021), and plays an important role during the metabolism of hepatocytes. It has been proven to be beneficial in treating a range of diseases, and is anticipated to be used for multiple medical‐related purposes in recent years (Kaneko et al., 2006; Maiese et al., 2009; Oblong, 2014; Stevens et al., 2007), and is widely available on the market at a reasonable price. The previous study revealed that NAM shows health improvement by reducing oxidative stress and inflammation (Mitchell et al., 2018) and may promote liver regeneration by activating sirtuin‐1 (SIRT1) (Wan et al., 2019). In addition to this, related studies showed that nicotinamide riboside (NR) (Wang et al., 2018) and nicotinamide mononucleotide (NMN) (Assiri et al., 2019), two other NAD^+^ precursors, have the potential to attenuate alcohol‐induced liver injuries. However, up to the moment, there are few research on the effect of NAM treating ALD, and the deeper connection between NAM and energy metabolism in ALD is yet to be explored.

In this bedside‐to‐bench study, we chose NAM to explore the impact of NAD^+^ supplementation on alcohol‐induced liver injury and its connection to energy metabolism regulation. The protective effect of NAM on ALD was examined. Subsequently, the energy‐producing organelle, mitochondrial structure, and function were tested. Moreover, to investigate the capacity of liver regeneration after NAM treatment, we also performed partial hepatectomy on mice fed with chronic‐plus‐binge ethanol (NIAAA model) (Bertola et al., 2013). The results confirmed our conjecture that by increasing NAD^+^ level with NAM, the alcohol‐induced energy supply disorder was reversed; and subsequently, liver regeneration and liver function were also improved effectively.

## 2 MATERIALS AND METHODS

### 2.1 Animal experiments

Male C57BL/6J mice, 9 weeks old, were purchased from SPF Biotechnology Co., Ltd. (Beijing, China). Mice were housed in colony cages with a 12‐h light–dark cycle in the temperature‐controlled environment, and randomly divided into four groups: control group (CTRL) treated with Lieber–DeCarli control liquid diet (Bio‐Serv, #F1259SP), ethanol‐treated group (EtOH) treated with Lieber–DeCarli ethanol liquid diet (Bio‐Serv, #F1258SP), ethanol‐treated with NAM‐supplied group (EtOH+NAM), and control treated with NAM group (NAM). After prefeeding with the control liquid diet for the first 5 days, mice in EtOH group and EtOH+NAM groups were fed with ethanol liquid diet for 10 days, while mice in CTRL and NAM groups were pair‐fed with control liquid diet. Mice in EtOH+NAM and NAM groups were given NAM (SIGMA, USA, #N0636) which was dissolved in purified water by oral gavage at a dose of 250 mg/kg BW once daily during the 10 days, mice in CTRL and EtOH groups were orally gavaged with the equivalent volume of purified water. Food consumptions were monitored daily to ensure enough ethanol intake. On day 11, mice were gavaged with a single dose of ethanol (5 g/kg BW) and euthanized 9 h later, their blood and liver samples were collected for further analysis.

To assess the liver regeneration, each group was assigned and treated as above, partial hepatectomy (PH) was performed 9 h after the ethanol gavage under anesthesia. Mice were housed in a sterile environment after PH, and blood and liver samples were collected on the first, fourth, and seventh days after PH.

All animal experiments were approved and performed according to the Guidelines of the Laboratory Animal Ethical Committee established by the Fifth Medical Center of Chinese PLA General Hospital, Beijing, China. The body weight and food intake condition were monitored every day during the experiments.

### 2.2 Biochemical assays

In serum, aspartate transaminase (AST), alanine aminotransferase (ALT), glutathione (GSH), malondialdehyde (MDA), gamma‐glutamyltransferase (GGT), and triglyceride (TG) were all detected with the corresponding biochemical kits from Nanjing Jiancheng Bioengineering Institute (Nanjing, China). Nicotinamide phosphoribosyltransferase (NAMPT) was determined by the ELISA kit from Jianglaibio (JL20484, Shanghai, China). In liver tissue, NAD^+^ was examined by the assay kit from Beyotime Biotechnology (s0175, Nantong, China) with WST‐8 method. IL‐22 was determined by the ELISA kit from Cloud‐Clone (SEC032Hu, USA). ATP level was measured with the ATP assay kit from Abcam (ab83355, Abcam Inc., Cambridge, MA, USA). GSH and oxidized glutathione (GSSG) were detected with the biochemical kits from Nanjing Jiancheng Bioengineering Institute (Nanjing, China). The activities of CS and SDH in liver were measured by corresponding activity assay kit (BC1060, BC0955, Solarbio, China). All the above analyses were performed following the manufacturer's instructions. Further details are provided in Supplementary Material.

### 2.3 Western blot analysis

Liver samples were homogenized in RIPA buffer with protease and phosphatase inhibitor cocktails. Equal amounts of proteins were separated on polyacrylamide gel and transferred to a nitrocellulose membrane. Target proteins were detected by western blot and immunostaining with specific primary antibodies, followed by related secondary antibodies. Beta‐actin antibodies and antibodies specific for CYP2E1 (ab28146), ADH1 (ab168748), ALDH2 (ab227021) were from Abcam, antibodies specific for SREBP‐1 (sc‐13,551), PPAR‐𝛾2 (sc‐390,740), SDH (sc‐390,381), CS (sc‐390,693), UCP2 (sc‐390,189), and NAMPT (sc‐393,444) were from Santa Cruz (Santa Cruz Biotechnology, Santa Cruz, CA, USA). Bands were visualized by ECL kit (Pierce, Rockford, IL, USA).

### 2.4 Transmission electron microscopy (TEM)

Liver tissues of 1 mm^3^ from the same anatomical locations were fixed in 2.5% glutaraldehyde in 100 mM sodium cacodylate, then washed, postfixed, dehydrated, and embedded in Epon 812 resin. Radom 100‐nm sections were cut and stained with Reynold's lead acetate and aqueous uranyl acetate. Images were studied with an H‐7500 transmission electron microscope (Hitachi, Japan).

### 2.5 Histology and immunohistochemistry

For H&E staining, fixed liver tissues were dehydrated using a serial alcohol gradient and embedded in paraffin, then sectioned at a thickness of 5 μm, followed by the standard methods including dewaxing, hydration, staining, dehydration, and sealing.

For Oil red O staining, fixed liver tissues were treated overnight with 15 and 30% sucrose solution successively. Then, tissues were embedded in optimal cutting temperature and sliced into 6‐ to 8‐μm sections by freezing microtome. Standard methods were followed afterward including washing, staining, differentiation, and sealing.

As for Ki‐67 immunohistochemistry, liver sections were incubated overnight with anti‐Ki67 antibody (Abcam, ab15580) at a dose of 1:100 after being blocked in 10% goat serum in PBS, followed by standard steps. Cells stained in the nucleus are the positive cells in the cell cycle.

### 2.6 Statistical analysis

All results were from three independent experiments. Values are shown as means ± standard error of the mean (SEM). Comparisons between two groups or multiple groups were analyzed by unpaired Student's *t*‐test or one‐way ANOVA. A value of *p* < .05 was considered to be statistically significant. All analyses were done by SPSS 26.0 (IBM, USA).

## 3 RESULTS

### 3.1 NAM alleviates liver injury in mice fed with chronic‐plus‐binge ethanol

**FIGURE 1:**
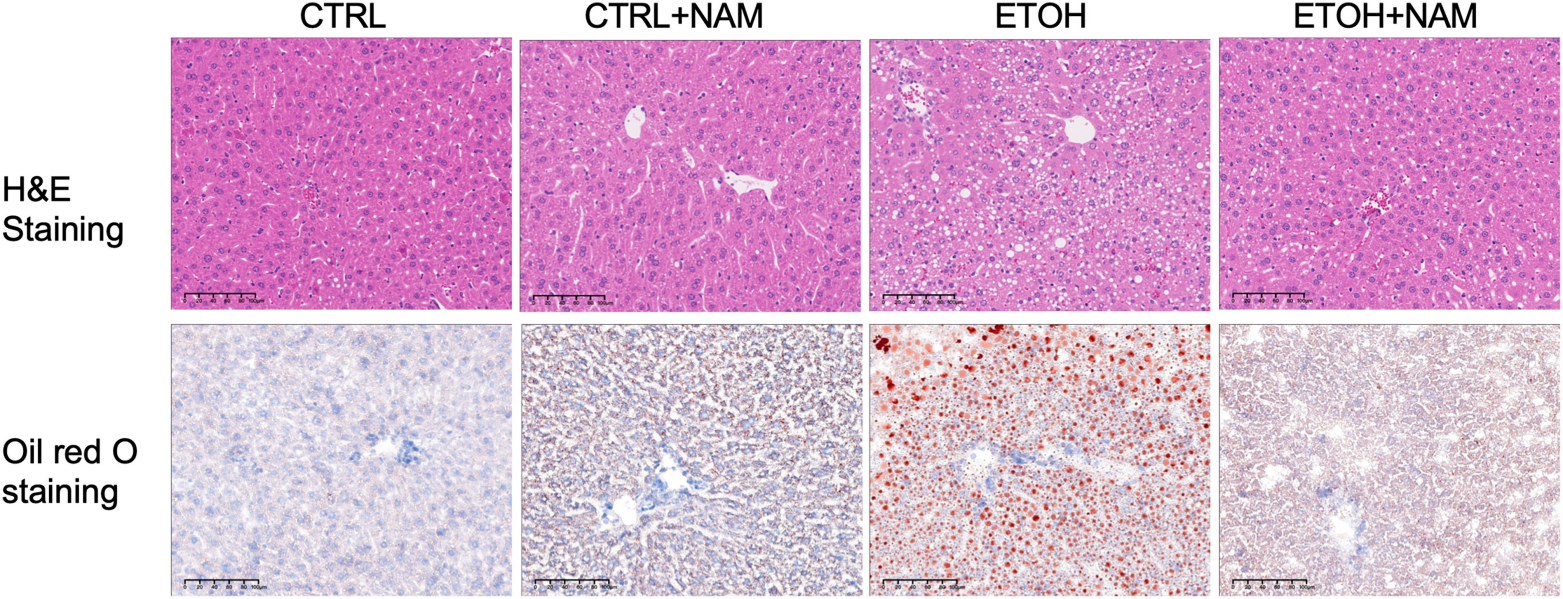
NAM alleviates hepatic steatosis in mice fed with chronic‐plus‐binge ethanol. Mice were treated with chronic‐plus‐binge ethanol feeding and NAM supplementation for 10 days. Representative images of mouse liver, H&E staining, and oil red O staining with 200 × magnification. NAM, nicotinamide.

To begin with, we assessed the histology alterations of NAD^+^ supplement in NIAAA mice. There were no statistical differences in baseline body weight among groups (Supplement Figure 1). The livers of the ethanol‐fed mice showed obvious microsteatosis according to histology results. In some ethanol‐fed mice, microsteatosis and inflammatory cell accumulation were also frequently observed. In contrast, alcoholic steatosis was completely abolished when the mice were given NAM in EtOH+NAM group (Figure 1).

**FIGURE 2:**
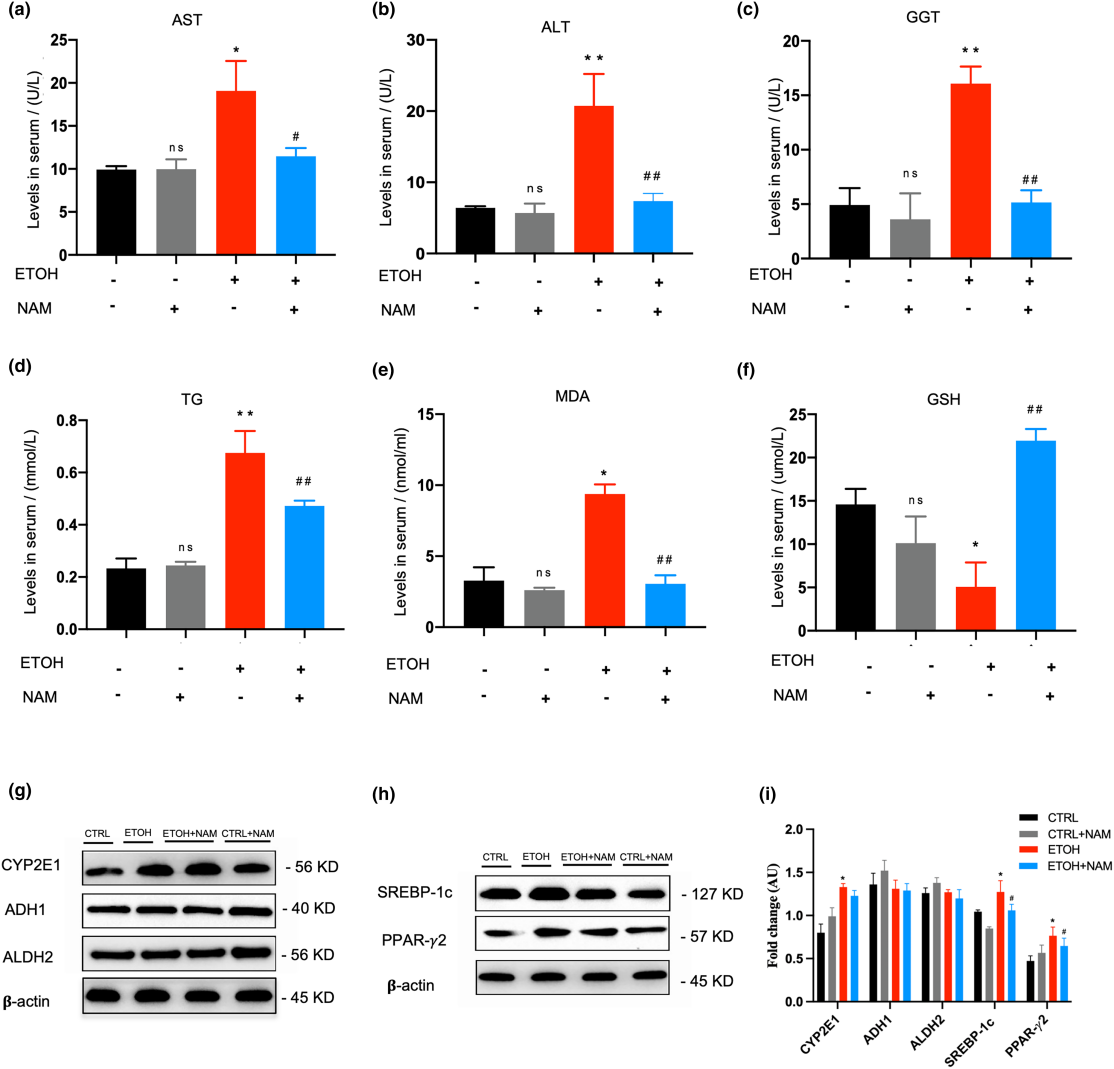
NAM alleviates hepatic steatosis in mice fed with chronic‐plus‐binge ethanol. Mice were treated with chronic‐plus‐binge ethanol feeding and NAM supplementation for 16 days. (a) Serum AST levels. (b) Serum ALT levels. (c) Serum MDA levels. (d) Serum GSH levels. (e) Serum GGT levels. (f) Serum TG levels. (g) Western blot analysis of alcohol metabolic enzymes (CYP2E1, ADH1, and ALDH2) in mouse liver. (h) Western blot analysis of lipid metabolic regulators (SREBP‐1c and PPAR‐γ2) in mouse liver. (i) Western blot fold changes of control. Results were expressed as fold changes of control. Data are expressed as mean ± SEM. *n* = 6–9/group, *p < .05 compared with the CTRL group; **p < .01 compared with the CTRL group; ^#^ *p* < .05 compared with the EtOH group; ^##^ *p* < .01 compared with the EtOH group. ADH1, alcohol dehydrogenase 1; ALDH2, aldehyde dehydrogenase 2; ALT, alanine aminotransferase; AST, aspartate aminotransferase; CYP2E1, cytochrome P450 2E1; GGT, γ‐glutamyl transpeptidase; PGC‐1α, peroxisome proliferator‐activated receptor γ coactivator‐1α; PPAR‐γ2, peroxisome proliferator‐activated receptor γ2; MDA, malondialdehyde; SREBP‐1c, sterol regulatory element binding protein‐lc; TG, triglyceride.

Biochemical assays were also performed. Ethanol consumption raised serum AST and ALT while NAM significantly decreased their values (*p* < .05) (Figure 2a,b). Serum GGT and TG also showed higher levels in EtOH mice than in CTRL mice. NAM markedly showed its effects by lowering the serum GGT and TG in EtOH+NAM group (*p* < .05) (Figure 2c,d). Oxidative stress was also elevated in the EtOH group, ethanol consumption upped MDA and drained GSH. NAM treatment saved this situation, the level of MDA and GSH showed no significant difference between EtOH+NAM group and the CTRL group (*p* > .05) (Figure 2e,f). NAM treatment in normal mice did not induce any liver injury in pathological or biochemical assays. All these biochemical results were consistent with pathological findings.

Major enzymes involved in the ethanol‐metabolizing pathways and key regulators in the lipogenesis signal pathway were also tested. The alcohol dehydrogenase1 (ADH1) and acetaldehyde dehydrogenase 2 (ALDH2) expression did not show any difference among all the groups (Figure 2g). Ethanol‐induced hepatic CYP2E1 expression, yet NAM did not make a difference in this. Nevertheless, sterol regulatory element binding protein‐lc (SREBP‐1c) and peroxisome proliferator‐activated receptorγ2 (PPAR‐γ2) were lowered by NAM supplement when they were elevated by ethanol (Figure 2h).

### 3.2 NAM supplement improves liver NAD ^+^ content in mice fed with chronic‐plus‐binge ethanol

**FIGURE 3:**
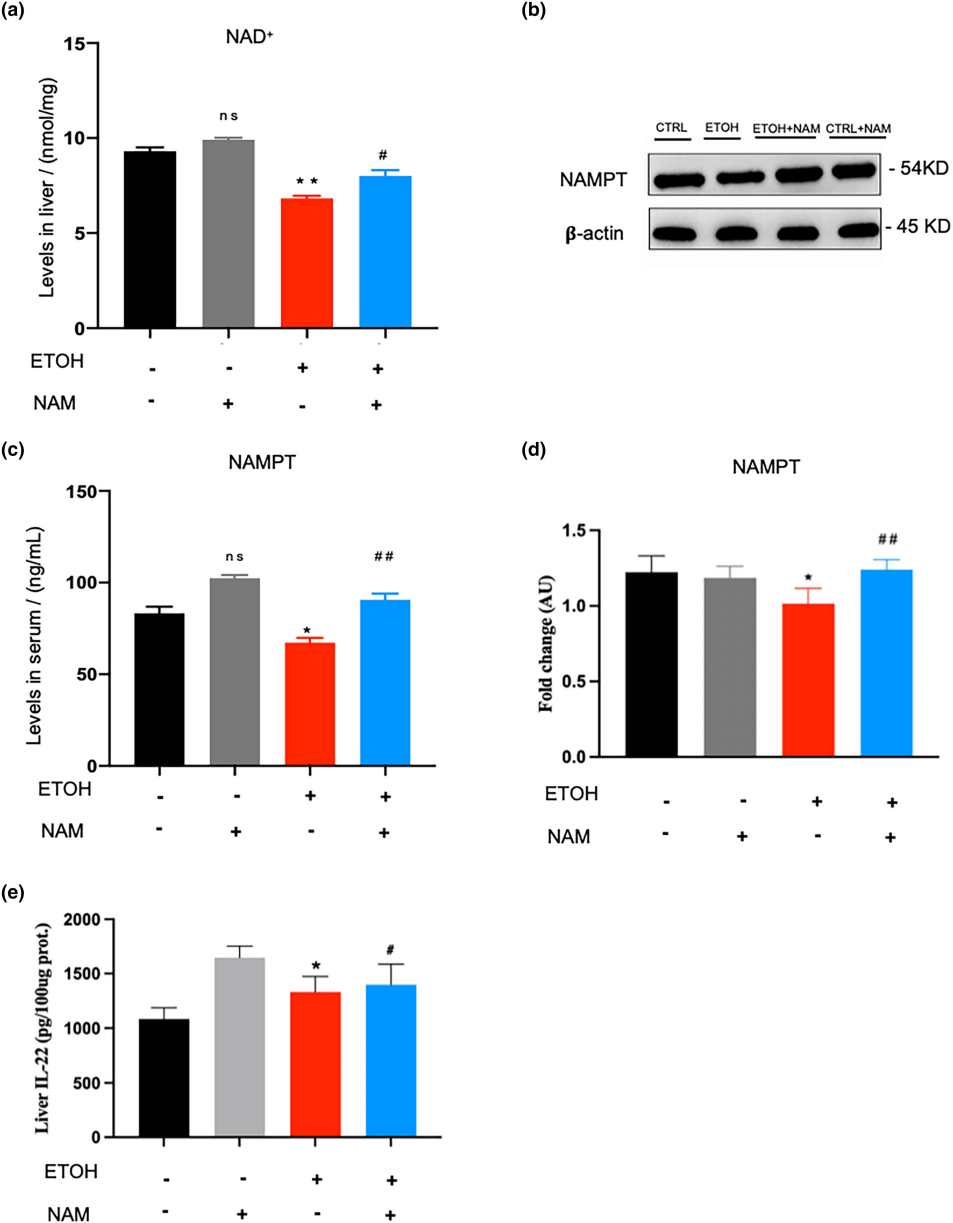
NAM significantly improves alcohol‐induced NAD^+^ drain. (a) Liver NAD^+^ levels. (b) Western blot analysis of NAMPT in mouse liver. (c) Serum NAMPT levels. (d) Western blot fold changes of control. (e) Liver IL‐22 level. Data are expressed as mean ± SEM. *n* = 6–9/group, *p < .05 compared with the CTRL group; **p < .01 compared with the CTRL group; ^#^ *p* < .05 compared with the EtOH group; ^##^ *p* < .01 compared with the EtOH group. NAD^+^, nicotinamide adenine dinucleotide; NAM, nicotinamide; NAMPT, nicotinamide phosphoribosyl transferase.

Following this, we examined the effect of NAM supplementation on the liver NAD^+^ pool and found that NAM significantly increased liver NAD^+^ content in NIAAA mice (*p* < .05). In detail, the NAD^+^ level in the CTRL group was 9.32 ± 0.19 nmol/mg, and it significantly decreased to 6.83 ± 0.14 nmol/mg in the EtOH group (*p* < .05). When the NIAAA mice were given NAM, the level of NAD^+^ increased to 8.01 ± 0.30 nmol/mg. There was no significant statistical difference in liver NAD^+^ levels between NAM and CTRL groups (Figure 3a).

NAD^+^ production is mainly associated with the NAD^+^ salvage pathway (Nakahata et al., 2009). So, we examined the expression of NAMPT, the key rate‐limiting enzyme in the NAD^+^ salvage pathway. Results showed that NAMPT expression was inhibited in EtOH mice (Figure 3b,c). Surprisingly, the NAM supplement reversed this situation and significantly enhanced the expression of NAMPT in the EtOH+NAM group. NAM supplement also increased the NAMPT levels in the liver and serum of normal mice (NAM group).

### 3.3 NAM prevents alcohol‐induced mitochondrial structure change

**FIGURE 4:**
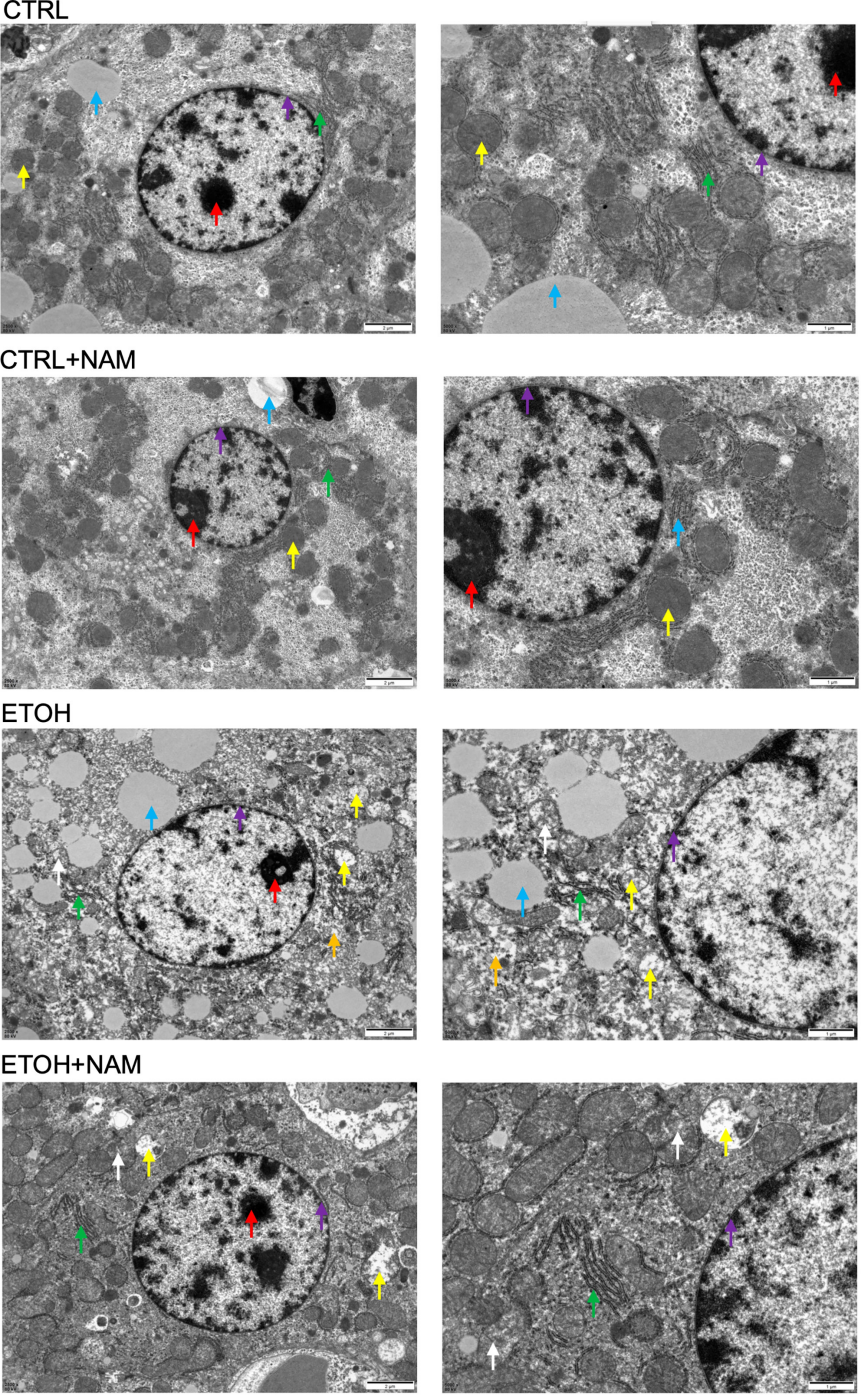
NAM improves the alcohol‐induced mitochondrial structure change. Representative liver electron micrographs. Chromatin (red arrow), nuclear membrane structure of the nucleus (purple arrow), intracytoplasmic lipid structure (blue arrow), intracytoplasmic mitochondrial membrane (yellow arrows), irregular cavity formed after suspected protein degradation (orange arrow), endoplasmic reticulum (green arrow), and intramitochondrial cristae (white arrow). NAM, nicotinamide.

NAD^+^ is the key driver of multiple energy metabolism pathways. The upper results show that the supplementation of its precursor NAM could prevent alcohol‐induced liver injury. The potential mechanism might be that NAM rescues energy metabolism disorder in mice fed with chronic‐plus‐binge ethanol. Therefore, the structural integrity of mitochondria, the energy‐producing organelles was examined through TEM assay. We found that the ultrastructure of mitochondria in the EtOH group was damaged, and abnormal structure exhibited: a small number of irregular cavities formed after suspected protein degradation was seen in the cytoplasm, as shown by orange arrows; a large number of mitochondria in the cytoplasm went swelling, and the matrix electron density was reduced and vacuolated, as shown by yellow arrows; some mitochondrial cristae structures were lost or broken, and incompletely damaged mitochondrial cristae were seen around the mitochondrial bilayer membrane, and the intramitochondrial cristae were disorganized, as shown by white arrows. By contrast, mitochondrial damage was well improved in the EtOH+NAM group. The mitochondrial density was slightly increased and mitochondrial swelling was reduced. Moreover, there was no vacuolar change or dissolution in the mitochondria in the EtOH+NAM group (Figure 4). NAD^+^ supplement in normal mice (NAM group) showed no obvious changes in mitochondrial structure, compared with that in the CTRL group.

### 3.4 NAM improves alcohol‐induced mitochondrial dysfunction

**FIGURE 5:**
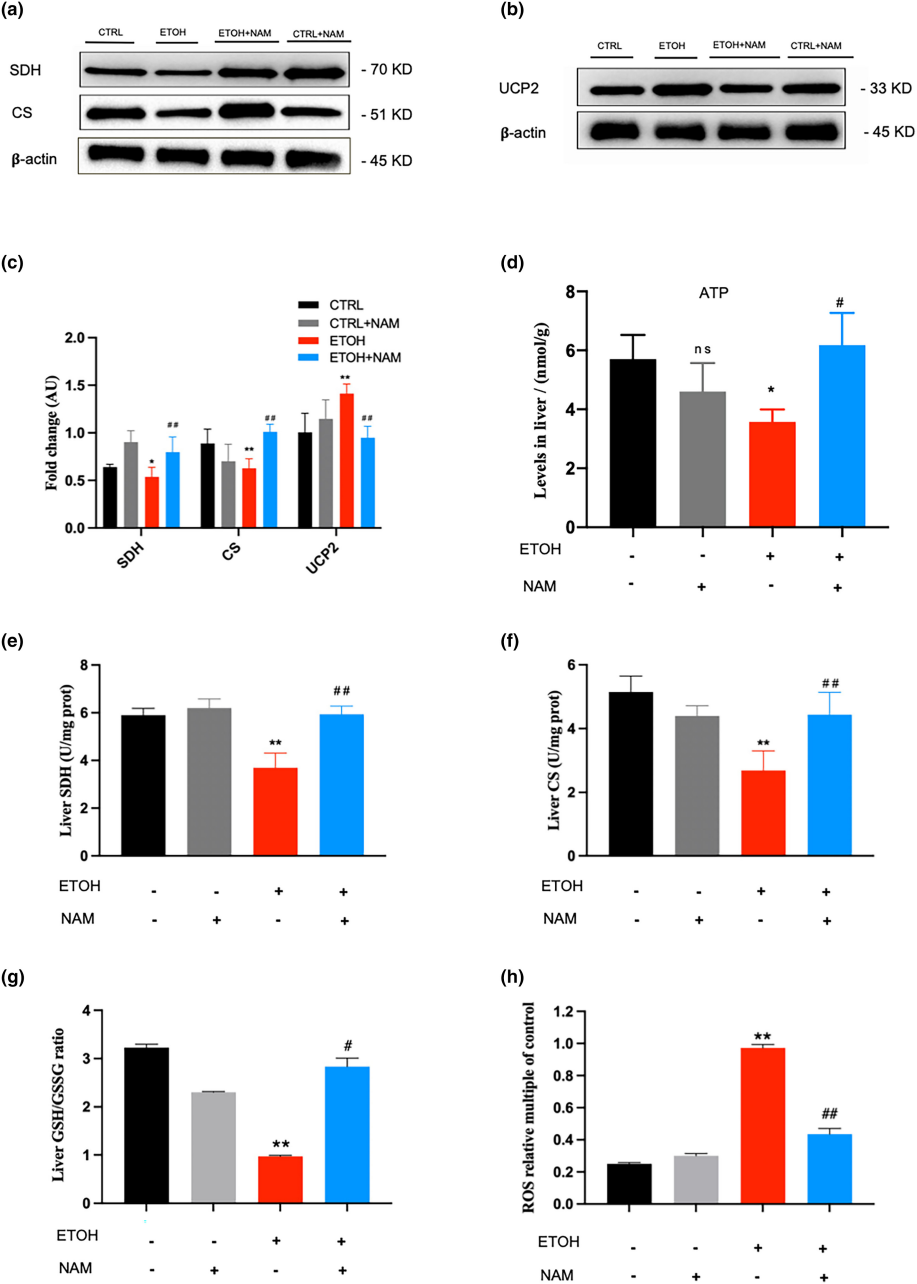
NAM significantly improves alcohol‐induced mitochondrial dysfunction. (a) Western blot analysis of SDH, CS in mouse liver. (b) Western blot analysis of UCP2 in mouse liver. (c) Western blot fold changes of control. (d) Liver ATP levels. (e) Liver SDH activity. (f) Liver CS activity. (g) Liver GSH/GSSG ratio. (h) ROS relative multiple of control. Data are expressed as mean ± SEM. *n* = 6–9/group, *p < .05 compared with the CTRL group; **p < .01 compared with the CTRL group; ^#^ *p* < .05 compared with the EtOH group; ^##^ *p* < .01 compared with the EtOH group. CS, citrate synthase; NAM, nicotinamide; SDH, succinate dehydrogenase; UCP2, uncoupling protein 2.

Given the above findings, exploring the effect of NAM supplement on alcohol‐induced mitochondrial dysfunction would be our next step. Succinate dehydrogenase (SDH) and citrate synthase (CS), two rate‐limiting enzymes in the tricarboxylic acid cycle (TCA) cycle, were tested (Figure 5a). Results revealed that SDH and CS were expressed significantly lower in the EtOH group than in the CTRL group, yet were shown as a normal level in EtOH+NAM group. Interestingly, uncoupling protein 2 (UCP2), a membrane protein that uncouples electron transport in the respiratory chain from ATP synthesis, was upregulated by EtOH intake, which is an indicator of the increased degree of oxygenation coupling, was lowered in the EtOH+NAM group, suggesting that the NAM supplement protects against EtOH‐induced UCP2 dysregulation.

ATP levels in the liver were also tested. Results showed that NAM rescues the low production of liver ATP in NIAAA mice. When given NAM, the liver ATP level in the NIAAA mice significantly raised (6.18 ± 1.09 nmol/g) when it was lowered in the EtOH group (3.58 ± 0.42 nmol/g) (*p* < .05). There was no significant difference in liver ATP levels between NAM and CTRL groups (Figure 5d).

### 3.5 NAM alleviates liver injury and motivates liver regeneration in mice fed with chronic‐plus‐binge ethanol after PH

Now we were shown that NAM supplement could prevent alcohol‐induced mitochondria structure and function disorder, as well as maintain the ATP levels in the liver. This result might suggest that liver self‐healing and damage prevention could benefit from NAM supplementation. To investigate the impact of NAM on the energy‐dependent process of liver regeneration in our mice, 40% PHx was carried out.

The histology results revealed that 1 day after PH, the ETOH group exhibited more serious liver injury and steatosis than the CTRL group, while NAM greatly alleviated liver injury and steatosis induced by PH in the ETOH+NAM group. Following 4 and 7 days post‐PH, the basic structure of liver cells in the ETOH+NAM group recovered faster than that in the ETOH group (Supplement Figure 2A).

**FIGURE 6:**
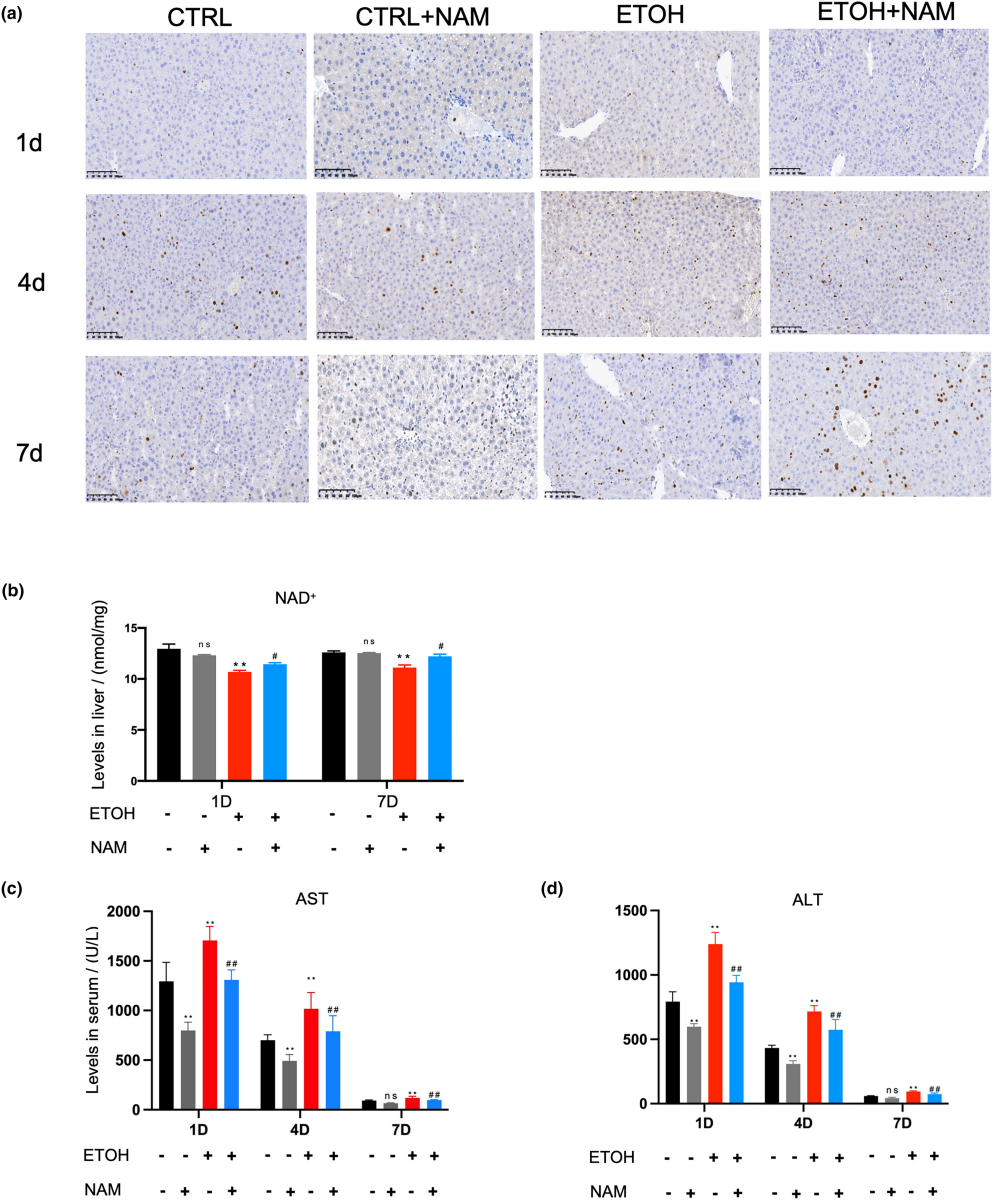
NAM motivates liver regeneration in NIAAA mice after PH. (a) Immunohistochemistry for Ki‐67 at the indicated time points. Black scale bars, 200 μm. (b) Serum NAD^+^ levels. (c) Serum AST and (d) ALT levels at the indicated time points following PH. Results were expressed as fold changes of control Data are expressed as mean ± SEM. *n* = 3–5/group, *p < .05 compared with the CTRL group; **p < .01 compared with the CTRL group; ^#^ *p* < .05 compared with the EtOH group; ^##^ *p* < .01 compared with the EtOH group. ALT, alanine aminotransferase; AST, aspartate aminotransferase; NAD^+^, nicotinamide adenine dinucleotide; NAM, nicotinamide; PH, partial hepatectomy.

The Ki‐67 immunohistochemistry staining was performed to validate the proliferation capacity of hepatocytes after PH (Figure 6a). At the fourth and seventh days post‐PH, we observed more increased active proliferation hepatocytes in the EtOH+NAM group than that in the EtOH group. These results suggested that NAM may accelerate ATP generation to support liver regeneration after PH. We also tested the serum AST, ALT, and liver NAD^+^ levels in each group. A major increase of AST and ALT levels in each group due to PH was observed, while that in the ETOH+NAM group were significantly lower than ETOH or CTRL group (*p* < .05). The AST and ALT values dropped back on the seventh day post‐PH (Figure 6c,d). NAD^+^ level showed a similar trend, it was significantly lower in the ETOH group than in the ETOH+NAM group at 1 and 7 days after PH (*p* < .05) (Figure 6b).

## 4 DISCUSSION

Our previous study suggested that metabolic disturbances related to cellular energy supply were the underlying mechanism for the development and progress of ALD (Cheng et al., 2022; Huang et al., 2021). As the key coenzyme, NAD^+^ plays a critical role in the TCA cycle and glycolysis process to regulate ATP production for normal physiological activity (Covarrubias et al., 2021; Verdin, 2015). However, during the process of ethanol metabolism, the liver NAD^+^ is largely consumed to oxidize ethanol to acetaldehyde and regenerate to acetic acid. Chronic ethanol intake and its metabolism could drain NAD^+^. This alternation of NAD^+^ content not only promotes alcohol‐induced liver injury but also limits liver regeneration capacity after injury. Accordingly, NAM supplement might be a potential way to improve the energy metabolism disorder caused by ethanol and maintain the liver regeneration capacity for the treatment or prevention of ALD.

In the present study, we mimicked the drinking pattern of ALD patients by applying a chronic‐plus‐binge ethanol‐feeding mice model. Results suggested that NAM supplements indeed improved alcohol‐induced liver injury and steatosis by regulating NAD homeostasis, protecting mitochondria from injury, activating the TCA cycle and ATP production, and maintaining the liver regeneration capacity. Ethanol taken induces CYP2E1 expression, which is an active producer of reactive oxygen species (ROS) and a promoter of alcohol‐induced oxidative liver injury (Leung & Lu, 2017). NAM supplementation inhibited enhanced expression of CYP2E1, and it might reduce the ethanol metabolism‐associated ROS accumulation and toxic oxidative adduct production to decrease hepatocyte injury and cell death. Our results also showed that NAM reduced liver MDA and increased reductants GSH in ethanol‐fed mice, indicating a decrease in ROS levels. Interestingly, there were no changes in CYP2E1, ADH1, and ALDH2 in NAM‐treated normal mice. Increased expression of SREBP‐1c and PPAR‐γ2 after EtOH treatment was also reversed by the NAM supplement, which could contribute to keeping the normal liver structure in the EtOH+NAM group. All these results imply that the process affected by exogenous NAD^+^ supplementation in EtOH‐treated mice was not only the metabolic process of alcohol in the liver but also the other related physical metabolism in vivo. It was known that alcoholic liver injury results in an imbalance of NAD^+^/NADH ratio and an obstruction of TCA due to NAD^+^ depletion, resulting in a reduced energy supply (Wang et al., 2018). Hence, the modification of energy production would be the key point of our following study.

NAM is reported to have the effect of antioxidant, anti‐inflammatory, antiapoptotic, and antiaging both in animal studies and clinical research (Hwang & Song, 2017; Maiese et al., 2009; Mitchell et al., 2018; Verdin, 2015). We examined the effect of NAM on the liver NAD^+^ pool as an NAD^+^ precursor and found that NAM supplement indeed increased liver NAD^+^ levels in the NIAAA model. The improvement might be associated with the NAD^+^ salvage pathway, with the evidence of enhanced expression of NAMPT, the key rate‐limiting enzymes in the NAD^+^ salvage pathway (Nakahata et al., 2009).

In light of that mitochondria are the main power packs for the synthesis of ATP (Maassen et al., 2002), we tested the impact of NAM on the mitochondrial structure and the related function subsequently. There are few studies focusing on the interaction between NAM and mitochondria, research showed that NAM enhances mitochondria quality through autophagy activation in human cells (Kang & Hwang, 2009), and protects retinal ganglion cells against mitochondrial and metabolic dysfunction (Tribble et al., 2021), yet the specific information is relatively poor. In this study, we found that NAM reversed the mitochondria structure damage and rescued the mitochondria membrane protein CS, SDH, and UCP2 expression in EtOH‐treated mice (Figures 4 and 5). NAM also promoted the ATP contents in EtOH‐treated mice. SDH activity can be used as an index to evaluate the running degree of the TCA cycle and is one of the markers of mitochondrial function (Kamboj & Sandhir, 2011; Medina‐Navarro & Guerrero‐Linares, 2009). CS, as the entrance to the TCA cycle, is regulated in a variety of ways to control the entry of other enzymes (Ge et al., 2015). CS and SDH are the key enzymes in the TCA cycle, and the upregulation of these two enzymes in the NIAAA model by NAM supplement indicated that NAM might promote the TCA cycle to restore ATP production and overcome the energy supply barrier by EtOH. Consistent with the degradation of ATP levels, significant UCP2 levels were found in the livers of ETOH mice. UCP2 is one of a family of conserved proteins located in mitochondrial intima that cause mitochondrial proton leakage (Zhao et al., 2019), high UCP2 expression leads to mitochondrial uncoupling and reduced membrane potential (Toda & Diano, 2014), resulting in reduced ATP synthesis. It could be seen that ethanol taken enhanced UCP2 expression while NAM represses this situation. It might imply that NAM inhibited ethanol‐induced mitochondrial uncoupling, reduced membrane potential, and promoted mitochondrial function as normal in the EtOH+NAM group. These results are in accordance with the transmission electron microscopy results.

Not just the ability of antiinjury, but the repair capacity is also critical to protect the liver from alcohol‐induced liver injury. It has been reported that NAM regulates the liver regeneration process and improves liver function (Wan et al., 2019). We performed PH on the NIAAA mice to explore this point. Our results demonstrated that NAM promoted hepatocyte proliferation, which in another word to say, liver regeneration, in addition, NAM raised liver NAD^+^ content post‐PH and alleviated liver injury.

## 5 CONCLUSION

Taken together, our study illustrated that NAM, a precursor of NAD^+^, could repair the structure and function of mitochondria to alter energy supply disorder in chronic‐plus‐binge alcohol‐feeding mice. This energy metabolism modification not only protects the liver from injury but also keeps the capacity of liver regeneration prepared for injured liver section repair. NAD^+^ might be a potential target to improve energy metabolism in ALD patients, and to release or even reverse ethanol‐induced liver injury. NAM, a kind of soluble vitamin B3, would be promising therapeutics for ALD based on the present data, the low cost, and the absence of side effects. Further studies are needed to propose a more accurate mechanism as to how NAM promotes energy metabolism, and hopefully clinical testing at an early date.

## AUTHOR CONTRIBUTIONS

**Yao Liu:** Data curation (lead); formal analysis (lead); investigation (lead); methodology (lead); software (lead); validation (lead); visualization (lead); writing – original draft (lead); writing – review and editing (lead). **Cheng Cheng:** Conceptualization (supporting); investigation (supporting); methodology (supporting); software (supporting); validation (supporting); visualization (supporting); writing – original draft (supporting); writing – review and editing (supporting). **Han Gao:** Investigation (supporting); methodology (supporting). **Xue‐jin Zhu:** Methodology (supporting); validation (supporting). **Xian He:** Methodology (supporting); validation (supporting). **Ming‐xi Zhou:** Methodology (supporting); validation (supporting). **Yuan Gao:** Methodology (supporting). **Ya‐wen Lu:** Methodology (supporting). **Xin‐hua Song:** Methodology (supporting). **Xiao‐he Xiao:** Methodology (supporting). **Jia‐bo Wang:** Conceptualization (lead); data curation (supporting); formal analysis (supporting); funding acquisition (lead); project administration (lead); resources (supporting); supervision (lead); writing – original draft (supporting); writing – review and editing (supporting). **Chun‐jun Xu:** Conceptualization (supporting); data curation (supporting); formal analysis (supporting); investigation (supporting); project administration (supporting); resources (supporting); writing – original draft (supporting); writing – review and editing (supporting). **Zhi‐tao Ma:** Conceptualization (supporting); formal analysis (supporting); investigation (supporting); methodology (supporting); project administration (supporting); resources (supporting); supervision (supporting); writing – original draft (supporting); writing – review and editing (supporting).

## CONFLICT OF INTEREST STATEMENT

The authors declare that they have no known competing financial interests or personal relationships that could have appeared to influence the work reported in this paper.

## Supporting information


Appendix S1.

## Data Availability

The data that support the findings of this study are available from the corresponding author upon reasonable request.
