# Supplementary material for: Restoring energy metabolism by NAD + supplement prevents alcohol‐induced liver injury and boosts liver regeneration
Source: Food Sci Nutr. 2024 Apr 22;12(7):5100–10. doi: 10.1002/fsn3.4159 (PMC11266918; doi:10.1002/fsn3.4159)
Supplement: Supplementary file 1 — Appendix S1. [file FSN3-12-5100-s001.docx]

**Supplementary Material**

**Methods**

**1. Measurement of serum GSH**

| Operation Procedure | | |
| --- | --- | --- |
|  | Standard tube | Test tube |
| 50μmol/L GSH standard (μL) | 10 | - |
| Sample (μL) | - | 10 |
| Reagent I (μL) | 100 | 100 |
| Reagent II (μL) | 10 | 10 |
| Mix and let stand at room temperature (25°C) for 2 minutes | | |
| Reagent III (μL) | 50 | 50 |
| Start timing while adding reagent III, shake the plate gently to mix the reagents thoroughly, place the plate in an enzyme marker and read the absorbance value at 405 nm at 30 seconds. The absorbance value (A1) was read at 405 nm for 30 seconds. (A1), leave it at room temperature (25℃) for 10 minutes, and read the absorbance value (A2) at 10 minutes 30 seconds. Take the absorbance value (A2) and calculate △A=A2-A1. | | |

**2. Preparation of Liver sample for NAD^+^ examination**

Collect fresh liver, wash with pre-cooled PBS on ice, weigh about 30 mg of liver sample, cut with scissors and place into a homogenizer with 400 μl of NAD^+^/NADH extract for homogenization on ice. Subsequently centrifuged at 12,000g for 10 mins at 4°C, the supernatant was taken as the sample to be tested.

**3. Preparation of Liver sample for ATP examination**

Collect 10 mg of liver tissue, wash in cold PBS. Homogenize tissue in 100 µL of ATP Assay Buffer with a Dounce homogenizer with 10-15 passes. Centrifuge the sample for 5 minutes at 4°C at 13,000 g, using a cold microcentrifuge to remove any insoluble material. Collect supernatant and transfer to a new tube on ice for further test.

**4.** **Preparation of Liver sample for SDH examination**

Weigh 0.1g of liver tissue, add 1mL of reagent I and 10μL of reagent II (provided in the kit), grind thoroughly with an ice bath homogenizer or mortar and pestle, centrifuge at 11000g for 10min at 4℃, remove the supernatant and place on ice for measurement.

**5.** **Preparation of Liver sample for CS examination**

Weigh 0.1g of liver tissue, add 1mL of extract and 10μL of reagent II (provided in the kit), and homogenize with an ice bath homogenizer or mortar and pestle. Centrifuge the homogenate at 600g for 5 min at 4°C. Transfer the supernatant to another tube and centrifuge at 11000g for 10 min at 4°C. Add 200 μL of Reagent I and 2 μL of Reagent II to the precipitate and mix well with repeated blowing for CS determination and for protein concentration determination.

**Figures：**

**Supplement fig. 1.** Mice bodyweight in each group.


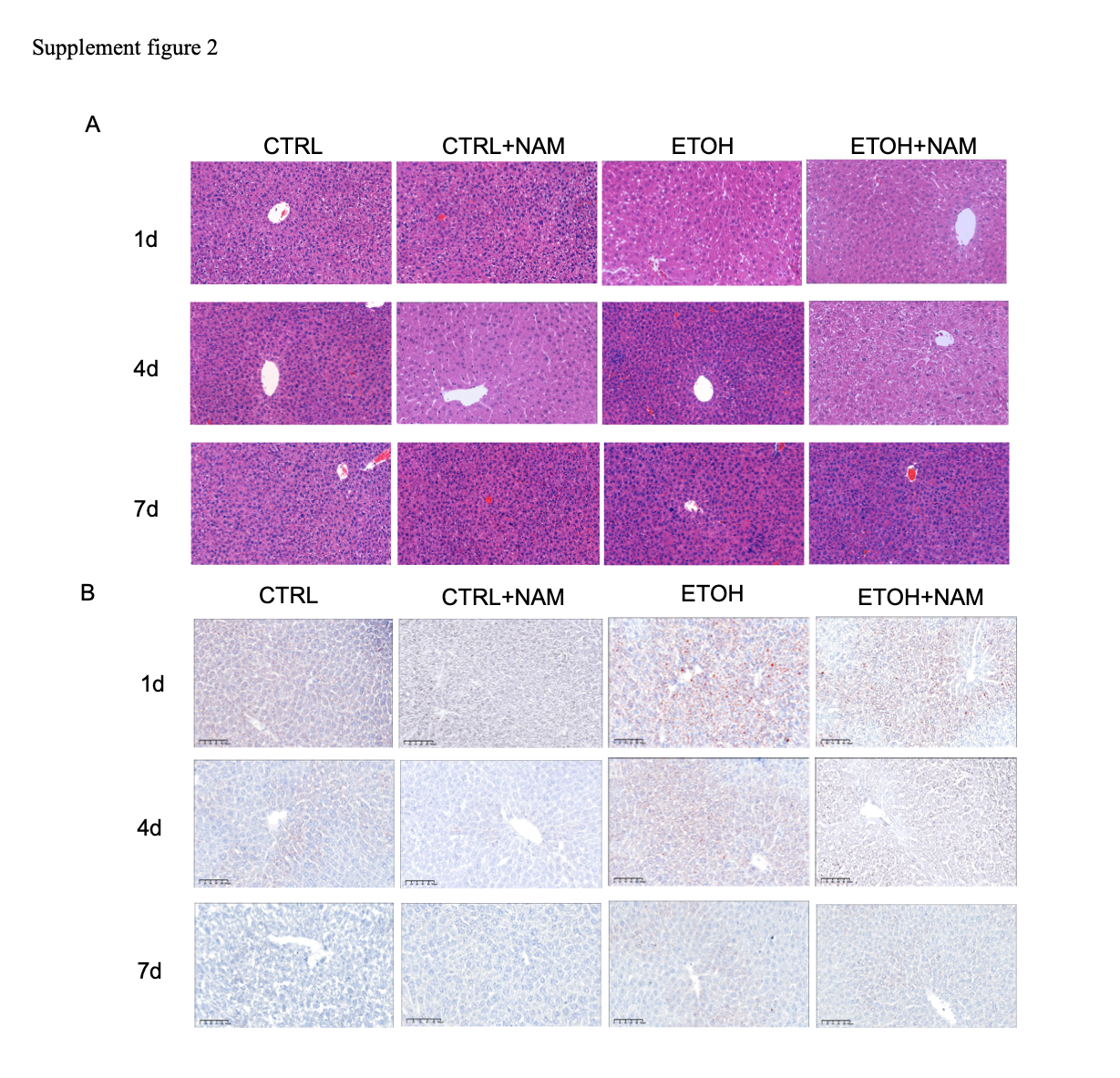


**Supplement fig. 2.** NAM alleviates liver injury induced by ethanol in mice after PH. (A) H&E staining and (B) oil red O staining demonstrated that PH livers staining with 200 × magnification.

Abbreviations: PH, partial hepatectomy; NAM, nicotinamide.
